# View from the top: Involvement of Namibia’s health ministry in laboratory quality improvement

**DOI:** 10.4102/ajlm.v3i2.195

**Published:** 2014-11-03

**Authors:** Mary N. Mataranyika, Landine K. Beukes

**Affiliations:** 1 Ministry of Health and Social Services, Namibia

## Introduction

Laboratories form the backbone of health systems, providing critical test results that aid diagnoses and identify the causes of disease outbreaks. Accurate laboratory results improve medical interventions, minimise surgical and treatment errors, decrease the length of hospital stays and reduce the cost of hospitalisation.^1^ Weak reporting systems and delayed or inaccurate processing of test results contribute to increased morbidity and mortality from major diseases of public health concern, such as tuberculosis, malaria, cholera and HIV.^2^

In 2012, Namibia’s Ministry of Health and Social Services (MoHSS), working with stakeholders from both the private and public sectors, developed Namibia’s first National Public Health Laboratory Policy^3^ and Strategic Plan^4^ (approved by the cabinet and launched in 2013) to guide the delivery of quality laboratory improvements and services. According to the Laboratory Policy, the MoHSS is mandated to offer high-quality laboratory services; support clinical care providers in disease treatment and prevention; provide disease surveillance and response; and develop policies that adhere to the national health principles of service equity, accessibility, affordability and sustainability.^3,4^ In order to address issues of poor reporting systems and inaccurate diagnostic results, the MoHSS provides oversight over the Namibia Institute of Pathology, the Blood Transfusion Service of Namibia and all private laboratories. The Government of Namibia endorses the World Health Organization (WHO) resolutions and programs aimed at laboratory improvements, including the 2008 Maputo Declaration to strengthen laboratory systems,^5^ the WHO Regional Office for Africa’s (AFRO) Stepwise Laboratory Quality Improvement Process Towards Accreditation (SLIPTA) framework^6^ and the Strengthening Laboratory Management Toward Accreditation (SLMTA) programme.^7^

## Implementing laboratory quality management in Namibia

In 2010, the MoHSS, with support from partners, began to implement the SLMTA programme and SLIPTA framework. The National Public Health Laboratory Policy encourages all laboratories to embrace SLMTA and SLIPTA, and a concerted effort is now being planned to assist the country's 64 laboratories to implement quality improvement. In June 2012, the MoHSS supported eight people from six Namibian laboratories to participate in a series of SLMTA workshops held at the Zimbabwe National Quality Assurance Programme in Harare, Zimbabwe. SLMTA in Namibia follows the standard three-workshop model, with improvement project implementation periods of approximately two to four months following each workshop.^7^

Progress of the enrolled laboratories is monitored through audits using the SLIPTA checklist. In addition to overall score and star ratings on a zero- to five-star scale, the checklist evaluates 12 quality areas. The African Society for Laboratory Medicine (ASLM) and the Clinical and Laboratory Standards Institute (CLSI) conducted official SLIPTA audits for four of the laboratories post-SLMTA; the remaining two laboratories did not receive audits because of lack of permission. One laboratory achieved three stars, two laboratories achieved two stars and one laboratory received zero stars. The highest scoring quality areas were client management, facilities and safety and purchasing and inventory. The lowest scores were observed in internal audit, management reviews and corrective action. Internal audits were either not being carried out or were conducted inconsistently. Management reviews did not address all appropriate topics and did not include action plans, responsibilities, timelines or status information. Most corrective actions and action plans were conducted by untrained staff.

### Lessons learned

To impact health outcomes and improve laboratory services, all stakeholders – including government agencies, funding bodies and staff – must be involved in and committed to achieving shared laboratory improvement goals. It is suggested that Ministries of Health adopt quality practices and standards as an integral part of their programmes and understand that external reviews and the accreditation of medical laboratories involve more than the mere assessment of conformance to standards for organisational processes.

In Namibia, there is a need for the MoHSS and the audited laboratories, with the assistance of ASLM, to jointly develop and follow up on a plan of action to address the gaps identified in the audits. Amongst the gaps in staff knowledge identified during these audits were how to manage non-conforming events; how to conduct internal audits and root cause analysis; and how to carry out preventive and corrective actions and monitor their effectiveness. In addition, staff lacked necessary skills in management of quality control and monitoring for trends, biases and shifts, as well as mulitirule (also called ‘Westgard rules’) violations and client/customer survey feedback. Nonconformities should be shared routinely with staff so that they can implement corrective actions. Furthermore, on-site quality assurance responsibilities for the Chief Medical Laboratory Technologists/Technologists in Charge were overloaded. Additional leaders should be trained in International Organization for Standardization (ISO) 15189:2012 standard requirements and management responsibilities.

It would be advisable for the MoHSS to develop a system of assessing all laboratories and addressing gaps identified, and then to use those assessments as a means of ensuring that quality becomes a condition for re-registration or licensing to operate a laboratory.

### Sustainability

An essential component in implementing a new programme is to plan for sustainability. The MoHSS is taking ownership of the quality improvement programmes by budgeting for future implementation of SLMTA and SLIPTA activities throughout the country. The MoHSS plans to build sustainability into its laboratory quality initiatives by increasing the number of in-country auditors, mentors and trainers, as well as by hosting training-of-trainers workshops so as to remove dependency on costly, external implementers. It is anticipated that this strategy will build capacity and promote self-sufficiency in Namibian medical laboratories. The MoHSS plans to build capacity for writing grant applications to support SLMTA and SLIPTA, trainings, operational research, dissemination of information and integration of all laboratory services with the aim of attaining accreditation to ISO standards in select laboratories. It would be advisable for the MoHSS to set up a national structure to audit and recognise laboratories; encourage development of innovative procedures and methods; allocate funding for equipment maintenance; and collaborate with accreditation bodies and other institutions in order to implement laboratory quality improvements.

As quality improvements become institutionalised in hospital laboratories, it is becoming evident that entire hospital systems are in dire need of similar quality improvement programmes. The Namibia MoHSS calls on international agencies to develop and adapt programmes such as SLIPTA and SLMTA for use throughout hospital systems so as to ensure continuous quality patient care.

## References

[1] Nkengasong JN, Nsubuga P, Nwanyanwu O, et al Laboratory systems and service are critical in global health: Time to end the neglect? Am J Clin Pathol. 2010;134(3):368–373. http://dx.doi.org/10.1309/AJCPMPSINQ9BRMU6 20716791 10.1309/AJCPMPSINQ9BRMU6 PMC7109802

[2] Peter TF, Rotz PD, Blair DH, et al Impact of laboratory accreditation on patient care in the health system. Am J Clin Pathol. 2010;134(4):550–555. http://dx.doi.org/10.1309/AJCPH1SKQ1HNWGHF 20855635 10.1309/AJCPH1SKQ1HNWGHF

[3] Namibia Ministry of Health and Social Services, National Public Health Laboratory Policy (establishing a strong public health laboratory system) [document on the Internet]. c2012 [cited 2014 Oct 14]. Available from: http://www.mhss.gov.na/files/downloads/dcc_3182_NPHL_policy_FINAL_new%20copy.pdf

[4] Namibia Ministry of Health and Social Services, National Public Health Laboratory Strategic Plan (establishing a strong public health laboratory system) [document on the Internet]. c2012 [cited 2014 Oct 14]. Available from: http://www.mhss.gov.na/files/downloads/687_3184_NPHL_STRATEGIC%20PLAN_FINAL_new%20copy.pdf

[5] World Health Organization Regional Office for Africa The Maputo declaration on strengthening of laboratory systems: [document on the Internet]. c2008 [cited 2013 Jan 02]. Available from: http://www.who.int/diagnostics_laboratory/Maputo-Declaration_2008.pdf

[6] World Health Organization’s Regional Office for Africa WHO guide for the stepwise laboratory improvement process towards accreditation in the African region (with checklist) [document on the Internet]. c2012 [cited 2014 Oct 20]. Available from: http://www.afro.who.int/en/clusters-a-programmes/hss/blood-safety-laboratories-a-health-technology/blt-highlights/3859-who-guide-for-the-stepwise-laboratory-improvement-process-towards-accreditation-in-the-african-region-with-checklist.html

[7] Yao K, McKinney B, Murphy A, et al Improving quality management systems of laboratories in developing countries: An innovative training approach to accelerate laboratory accreditation. Am J Clin Path. 2010;134(3):401–409. http://dx.doi.org/10.1309/AJCPNBBL53FWUIQJ 20716796 10.1309/AJCPNBBL53FWUIQJ

